# A Case Report of Carcinosarcoma of the Tongue Mimicking a Fibroma: An Enigmatic Lesion With a Diagnostic Dilemma

**DOI:** 10.7759/cureus.28203

**Published:** 2022-08-20

**Authors:** Aishwariya Mohanty, Susil Sahoo, N. C. Sangamesh, Abikshyeet Panda, Pallavi Mishra

**Affiliations:** 1 Department of Oral Pathology, Kalinga Institute of Dental Sciences, Kalinga Institute of Industrial Technology (KIIT) Deemed to be University, Bhubaneswar, IND; 2 Department of Oral Medicine and Radiology, Kalinga Institute of Dental Sciences, Kalinga Institute of Industrial Technology (KIIT) Deemed to be University, Bhubaneswar, IND

**Keywords:** oral cavity, fibroma, cacinosarcoma, tongue, spindle cell carcinoma

## Abstract

Carcinosarcoma of the tongue is a rare biphasic tumor composed of squamous cell carcinoma (SCC), either in situ and/or in invasive form, and a mesenchymal component but of epithelial origin. It is important to diagnose this variant because of its aggressive nature and tendency to metastasize early. The present report describes the case of a carcinosarcoma of the tongue in a 48-year-old male with a short history of 30 days, the clinical feature of which resembles that of an irritational fibroma. The diagnosis often represents a clinicopathological challenge where the study with immunohistochemical technique (IHC) is key to the histopathological diagnosis. We here present a case report of this rare tumor, with an unusual presentation, to contribute in part to better understanding and awareness of this rare malignancy.

## Introduction

Carcinosarcoma is a biphasic, poorly differentiated variant of squamous cell carcinoma (SCC) composed of carcinomatous and sarcomatous elements, but of epithelial origin. It was first described by Virchow in 1865 [1]. The World Health Organization classified this disease entity as malignant surface epithelial tumors of squamous cell carcinoma (SCC) and termed it "spindle cell squamous cell carcinoma (SC-SCC)" in its classification of tumors of the oral cavity and oropharynx [2]. It occurs most commonly in the upper aerodigestive tract, chiefly in the larynx, hypopharynx, esophagus, trachea, and its occurrence in the oral cavity is rare [3,4]. In addition, there is only a handful of literature outlining the location of this tumor in the tongue [5,6]. We here report a case of carcinosarcoma of the tongue with an unusual clinical presentation mimicking that of a fibroma.

## Case presentation

**Figure 1:**
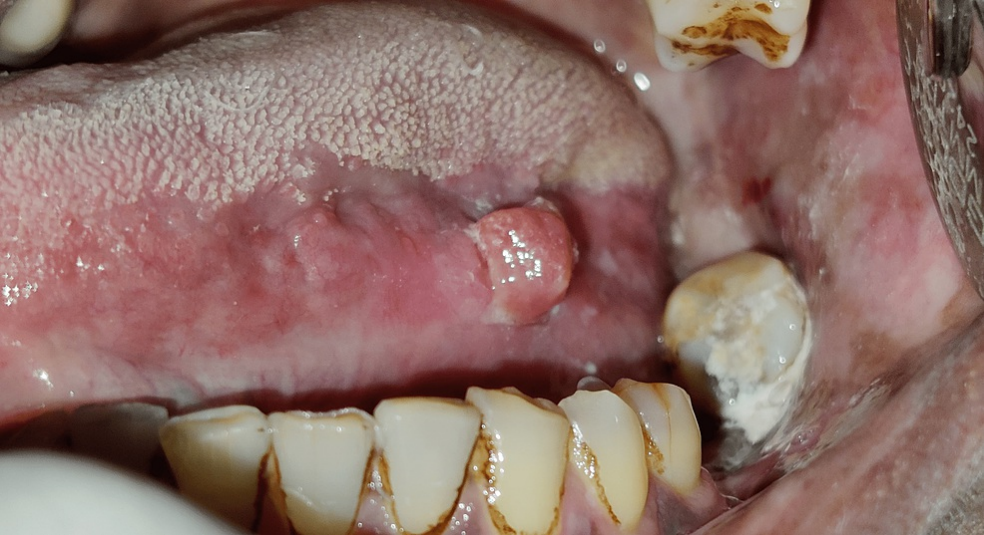
Intraoral photograph showing a well-defined sessile swelling in the left lateral border of the tongue.

A 48-year-old male patient was reported to the outpatient department with the chief complaint of growth on the left lateral border of the tongue for the past one month. The swelling was of gradual onset, slowly progressive, and was not associated with any pain or discharge. The patient had a habit of chewing a smokeless form of tobacco for the last 15 years. The patient doesn’t reveal any history of smoking, alcohol, or exposure to radiation. A clinical examination revealed a well-circumscribed sessile growth measuring about 1 cm × 1 cm in dimension on the left lateral border of the tongue. The growth appears to fit into the area of the missing tooth 36. The mucosa over the swelling appears normal and matches the adjacent mucosa (Figure 1). There was chronic irritation on the affected side of the tongue from the lower left third molar. Indentations of the teeth on the left lateral border of the tongue were also noted. There are also diffuse areas of depigmentation and hyperpigmentation on the left buccal mucosa. On palpation, the lesion was firm in consistency with a smooth surface. There was no pus discharge, bleeding, or any other associated symptoms of provocation like pain. The patient denied any history of tenderness or paraesthesia. No regional lymphadenopathy was noted clinically. The extensive clinical examination led to a provisional diagnosis of traumatic fibroma and differential diagnosis of pyogenic granuloma, hemangioma, and lipoma. Because the patient was an otherwise healthy man with an asymptomatic lesion, the clinical features of which resembled traumatic fibroma, a neoplastic condition was ruled out, and a benign condition was preferred.

**Figure 2:**
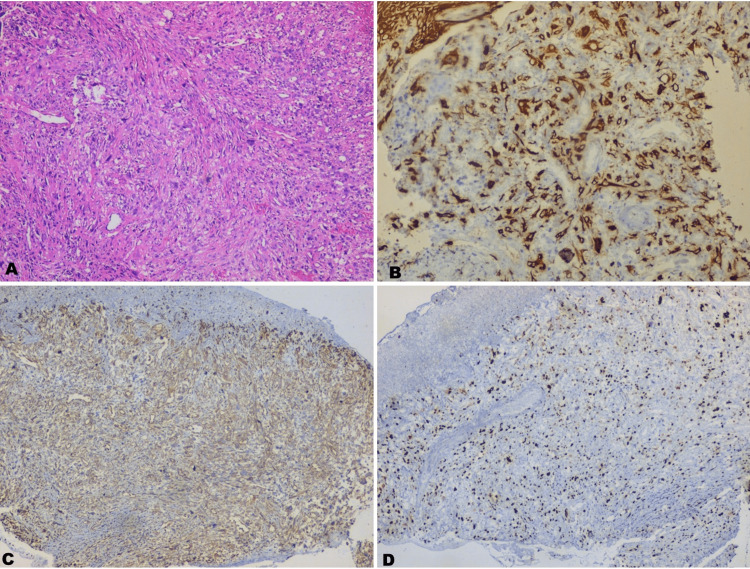
Histopathology and immunohistochemistry. (A) H&E stained section showing spindle cells arranged in a vague storiform pattern (×100 magnification). (B) IHC showing a carcinomatous component positive for cytokeratin. (C) IHC showing sarcomatous components positive for vimentin. (D) High proliferative Ki-67 labeling index. IHC: immunohistochemical technique.

The patient was advised to have routine blood investigations and a biopsy. Blood reports were within normal range, so an excisional biopsy was performed assisted with a laser and the H&E stained section showed ulcerated epithelium and an underlying loose fibro cellular connective tissue component with diffuse infiltration of undifferentiated malignant cells showing dysplastic features. At a few foci of dense cellular area, the spindle-shaped cells were arranged in short intersecting fascicles, usually forming a loose crisscross pattern (Figure 2A). Hence, a histopathological diagnosis of “undifferentiated neoplasm” was given and immunohistochemistry was recommended. The malignant cells were positive for both cytokeratin and vimentin, which confirmed that the lesion was composed of carcinomatous and sarcomatous components (Figures 2B, 2C). The immunoreactivity for Ki-67 was detected to establish the proliferative activity of the malignant cells, and the Ki-67 labeling index was found to be 25-50% (Figure 2D). This helped us to reach a final diagnosis of carcinosarcoma.

**Figure 3:**
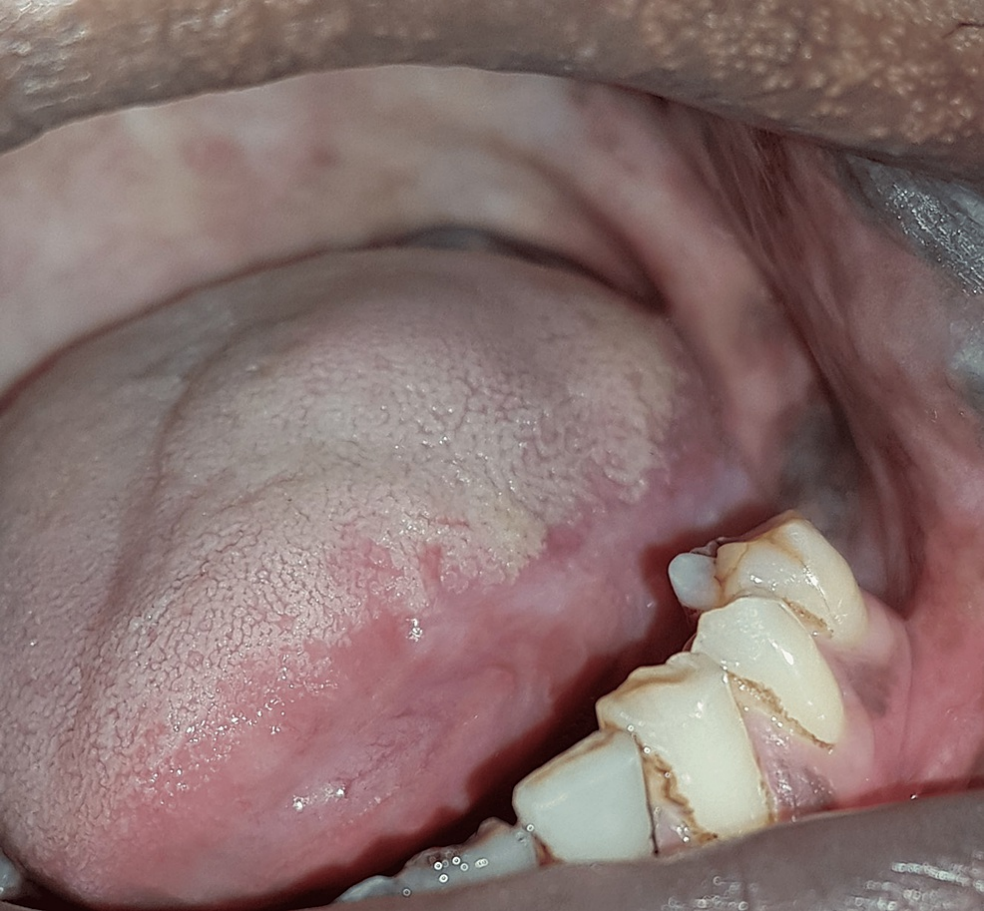
Post-operative follow-up photograph after two months.

The patient was referred to the department of oncology for further treatment where he had undergone 10% resection of the tongue, which included removal of the base and margins. The left submandibular lymph node resection was also done according to the surgeon’s decision. Histopathological reports revealed that the base, margins, and submandibular lymph nodes were free from malignant cells. The patient did not require any chemotherapy or radiotherapy and was prescribed antibiotics, analgesics, and multivitamins. After two months of follow-up, the patient reported with good prognosis and primary wound healing (Figure 3).

## Discussion

Carcinosarcomas are biphasic tumors demonstrated to be the monoclonal dedifferentiated types of conventional squamous carcinomas and true examples of epithelial-mesenchymal transition [4]. There is a great dispute among pathologists regarding the origin of this tumor. Due to disagreements regarding the origin of the tumor, carcinosarcoma has been designated by various names such as spindle cell squamous cell carcinoma, polypoid squamous cell carcinoma, sarcomatoid squamous cell carcinoma, sarcomatoid carcinoma, Lane tumor, collision tumor, pseudosarcoma, and pseudosarcomatous carcinoma [5]. This indicates the conflicting explanation of the histogenesis of the spindle cell component. Three main theories have been proposed to explain the histogenetic nature of spindle cells. The first theory explains the simultaneous emergence of spindle cells and epithelial cells from separate stem cells, justifying the name “collision tumor.” The second theory illustrates that the spindle cell component is due to the atypical reactive proliferation of the stroma, deserving the name “pseudosarcoma.” Finally, the third theory portrays that both epithelial and spindle cell components have the same monoclonal origin and “dedifferentiation” or “transformation” to spindle cells has occurred. However, recently, the monoclonal hypothesis has been widely accepted and is strongly supported by some studies [6].

The clinical picture as seen in the literature is reported between the sixth and seventh decade of life, with predominance in male adults [6]. The present case was reported in a 48-year-old male in the left lateral border of the tongue. In literature as well as in the current case, a history of predisposing factors like smoking, alcohol, and chewing tobacco is observed. Clinically, these tumors present as exophytic, polypoid, and ulcerated masses in the tongue and rarely as a flat lesion [6]. In our case, the clinical presentation was small, sessile swelling with a history of one month and mimicking that of a fibroma. The duration of symptoms ranged from 20 days to two years, with less than one year in 95% of patients [6]. The size mentioned in the literature ranges from 2.0 cm × 1.0 cm to 6 cm × 6 cm [7,8]. But in the current case, the size was 1 cm × 1 cm representing the smallest of all the spindle cell carcinomas (SpCCs) of the tongue reported in the literature. The lesion may be associated with other complaints like pain, dysphagia, odynophagia, and bleeding, which were not evident in our case [9].

The presence of both carcinomatous and sarcomatous components is the hallmark of this tumor. The carcinomatous component may be present as carcinoma in situ or an invasive SCC among the mesenchymal component, as was evident in this case. The sarcomatous component can be very monotonous, resembling granulation tissue, or may be composed of highly pleomorphic and undifferentiated cells that may exhibit elements such as myxoid stroma, cartilage, osseous, or proliferating spindle cell components [10]. The histopathological distinction between carcinosarcoma and malignant connective tissue tumors is sometimes difficult owing to the presence of anaplastic cells. In our case, it was essential to perform an immunohistochemical panel to establish the diagnosis of carcinosarcoma. Immunohistochemistry has a vital role in confirming the origin of cells. Markers like epithelial membrane antigen (EMA), high-molecular-weight cytokeratin (HMWCK), p63, CD10, smooth muscle actin (SMA), calponin, desmin, S100 protein, and HMB45 are also used to determine the origin [7]. In the present case, the positivity of cytokeratin and vimentin confirmed the epithelial and mesenchymal origin of cells. The differential diagnosis may be difficult in cases where the spindle cell component forms the majority of the tumor. SpCC can mimic true fibrosarcoma, but fibrosarcomas are uncommon in the head and neck. The presence of malignant squamous cells and immunohistochemical markers indicates that the diagnosis is correct. The spindle cell lesion must also be distinguished from malignant fibrous histiocytoma (via cytologic pleomorphism and multinucleate giant cells), rhabdomyosarcoma (via tadpole or strap cells), synovial sarcomas (via the age of presentation, location, and chromosomal translocation), and malignant peripheral nerve sheath tumors (showing nerve coursing of the tumor cells and herniation of tumor in blood vessels) [11].

Carcinosarcoma is an aggressive variant of squamous cell carcinoma, the prognosis of which is controversial and closely related to the depth of invasion and metastases, which are very common [8,10]. Also, carcinosarcoma of the tongue can occur due to metastasis of other metastatic malignant tumors. The tongue is the second most frequent site of metastasis in the oral mucosa after gingiva and represents approximately 28% of all malignant metastatic tumors of the oral mucosa [7]. Hence, an examination of the whole body should be done for the presence of any neoplasm and a long-term periodic follow-up is necessary.

To control both local and distant recurrence, surgical resection with neck dissection is widely regarded as the best treatment option in the oral cavity [5]. In terms of prognosis, surgical intervention, with or without radiotherapy, outperformed radiotherapy alone. The role of chemotherapy in the treatment of SC-SCC is not addressed [5]. Our patient had a 10% tongue resection, and his condition was closely monitored, with no recurrence to date.

## Conclusions

Carcinosarcoma constitutes a remarkable diagnostic challenge with prodigious histological and immunohistochemical overlap with other spindle cell tumors due to its heterogeneous nature. Because of its aggressive nature, recurrence, and early metastasis, this variant of SCC must be diagnosed and investigated. This case represents the smallest carcinosarcoma of the tongue reported so far in the literature. Hence, even the smallest of tumors should not be neglected and should be addressed with the utmost vigilance.
